# Torsional strength and deformation characteristics of HSC deep beams reinforced with cross-inclined stirrups

**DOI:** 10.1038/s41598-026-54158-z

**Published:** 2026-06-04

**Authors:** Rehab Fawzi, Hamed S. Asker, Ahmed M. Yousef

**Affiliations:** 1 Department of Civil Engineering, Delta University for Science and Technology, Mansoura, Egypt; 2 Department of Structural Engineering, Mansoura University, Mansoura, Egypt

**Keywords:** Cross-inclined stirrups, Deep beams, Torsion, Torsional deformation characteristics, High-strength concrete (HSC), Engineering, Materials science

## Abstract

The findings of this paper provide essential knowledge for the effective and economical design of deep beams subjected to pure torsion. As a result, this paper studies the torsional behavior of nine high-strength concrete (HSC) deep beams reinforced by cross-inclined stirrups. The test parameters comprised stirrup type (conventional stirrups and cross-inclined stirrups), inclination angle (45 and 60 degrees), stirrup spacing (100, 150, and 200 mm), the main longitudinal bars (4∅12 and 4∅18), and the side longitudinal bars (without and 4∅12). Torsional moment capacity, crack pattern, twisting angle, strain on longitudinal and transverse reinforcement, and torsional deformation characteristics in terms of stiffness and ductility were used to evaluate the tested beams’ torsional response. Test results clearly indicated that using the cross-inclined stirrup reinforcement improved the evaluated deep beams with conventional stirrups’ torsional capacity, stiffness, ductility, and overall performance. Reducing stirrup spacing from 200 to 100 mm increased torsional capacity by 49% for conventional stirrups and 65% for cross-inclined stirrups. An ABAQUS three-dimensional finite element model was built to evaluate the model’s ability to replicate the experimental beams’ torsional behavior. A theoretical model for ultimate torque calculation was also created. Compared to experimental findings, numerical and theoretical investigations were satisfactory.

## Introduction

Historically, torsion in reinforced concrete (RC) structures is commonly neglected, and its impacts was included in the safety factor instead of conventionally designed structures, resulting in brittle shear-torsion failures. As a result, torsional strength is essential in current designs, especially in complex structures like curved girders, spiral staircases, and beams subjected to eccentric or dynamic loads^1–5^. Large structures such as bridges, offshore constructions, and multistory buildings frequently utilize deep beams as structural components. They stand out due to their significant depth relative to their span and their narrow width. According to ACI 318–2025^6^ and ECP 203–2020^7^, deep beams have spans up to four times their depth or concentrated load regions within twice the depth from supports. Despite their increasing use in modern structures, research into their torsional behavior is limited, and current standards, such as the ACI 318–2025^6^, do not provide specific requirements for deep beams under torsional loads, despite their significance. For instance, transfer girders with significant eccentricity in multistory structures produce torsional moments due to offset load paths^8^. Alongside, curving bridge girders and edge beams in bridge engineering often experience torsion owing to load eccentricity produced by geometry, especially under asymmetric traffic conditions^9,10^. Similarly, ring deep beams in circular tanks or domes undergo torsion as a principal effect resulting from circumferential load distribution^11^.

Deep beams, unlike shallow beams, generate nonlinear strain distributions, rendering common stress analysis methods ineffective^12–14^. Yousef et al.^15^ asserted that span-to-depth ratios led to varying crack patterns, stress distributions, and torsional stiffness. The deep beam with an L/h ratio of 1 had an ultimate torque 54% greater than that of the beam with an L/h ratio of 4, despite equivalent reinforcing. In addition, deep beams demonstrate high torsional resistance to cracks compared to shallow beams, since a reduction in the L/h ratio limits crack formation. In other words, the crack width of shallow beams expands and propagates more significantly throughout the span. Moreover, deep beams demonstrated enhanced torsional stiffness after cracking due to their geometric configuration, unlike shallow beams. Consequently, further research into the torsional behavior of deep beams is necessary. 

The ultimate strength of reinforced concrete elements subjected to torsion can be predicted using two main theories. Skew bending theory served as the foundation for the old building codes from 1971 to 198^16,17^, whereas the new building codes have been based on truss model theory since 1995. The space truss analogy theory describes reinforced concrete beams under torsion in two phases: before cracking, the concrete section behaves like a thin-walled tube, with constant shear flow resisting the torsional moment. After cracking, the beam is modeled as a space truss, with inclined concrete strips handling compression and longitudinal bars and stirrups handling tension to resist the torsional forces^18–20^.

Although considerable research has been performed to improve the torsional performance of reinforced concrete beams, the majority of studies have concentrated on conventional vertical stirrups in shallow beams, employing advanced materials such as Ultra-High-Performance Concrete (UHPC) and Ultra-High Performance Fiber Reinforced Concrete (UHPFC), which have augmented cracking resistance and post-cracking behavior through fiber bridging mechanisms^21,22^. Externally bonded and internally reinforced FRP systems enhance torsional resistance of UHPC tubular beams by confining and distributing stress^23,24^. Hybrid strengthening methods such as FRP with strain-hardening cementitious composites (SHCC) or fiber meshes have improved ultimate capacity, stiffness, and energy absorption^25^. These advanced methodologies enhance torsional performance, although cost may limit their use.

According to previous studies, the use of High-Strength Concrete (HSC) beams generally exhibits higher torsional strength and cracked stiffness compared to normal-strength concrete (NSC) beams^26–28^. According to Ibrahim T. Mostafa et al.^29^, when the web reinforcement ratio and configuration are identical, the HSC specimens exhibit greater torsional strength and stiffness both before and after cracking compared to the NSC specimens. A. M. Yousef^30^ conducted experiments on deep beams that responded to pure torsion and discovered that enhancing the concrete compressive strength from fcu = 35.1 MPa to 80.7 MPa significantly boosts the observed cracking torsional strength by approximately 44% and marginally increases the ultimate torsional strength by around 20%.

Whilst other studies highlighted the benefits of employing spiral stirrups as torsional reinforcement in place of closed stirrups^31,32^. This method is particularly valued for its cost-effectiveness compared to other approaches, such as those involving steel fibers, glass fiber reinforced polymer sheets, and external prestressing techniques. A. Ibrahim et al.^33^ studied the torsional behavior of concrete beams reinforced with inclined spiral stirrups. The findings indicated that these stirrups improved the torsional capacity by approximately 16% compared to conventional closed stirrups. Additionally, the study revealed that this method resulted in greater twist angles, more ductile failure, and an increase in strain energy by about 27%. These findings align with Chalioris and Karayannis’s^34^ conclusions that rectangular spiral stirrups enhance the torsional capacity of RC beams. Additionally, locked rectangular spirals further enhanced the torsional strength of RC beams.

Implementing spiral stirrups in deep beams is challenging due to their geometric shape. Consequently, this study’s novelty is in demonstrating the impact of an affordable alternative method using high-strength concrete (HSC) and cross-inclined stirrups on deep beams under pure torsion, which has been little addressed in previous studies. The experimental data gathered were subsequently utilized to validate a numerical model created using the ABAQUS simulator. This model is intended to expand the experimental research by broadening the experimental parameters, thereby achieving a thorough comprehension of the torsional behavior of deep beams.

## Experimental program

### Material properties

For all deep beams, ready-mix of High-Strength Concrete (HSC) was used. The used mix proportions are selected according to ACI 211.4R^35^ and closely align with previously documented high-strength concrete compositions^36,37^. The HSC mix proportions, by weight per 1.0 m, were 495 kg ordinary portland cement, 1150 kg gravel, 580 kg natural sand, 154 kg water, and 55 kg silica fume. A superplasticizer with 2.2 percent binder weight was added. The mix achieved a cube compressive strength (f_cu_) of 91.7 MPa after 28 days, based on a mean of six cube specimens (150 × 150x150 mm) tested according to ASTM C39/C39M^38^. The average splitting cylinder tensile strength of six cylinder specimens (150 × 300 mm), tested according to ASTM C496/C496M^39^, was 5.8 MPa. The diameters of steel bars used in the test beams were 8 mm, 12 mm, and 18 mm. Table 1 lists steel bar mechanical properties.

**Table 1:** Mechanical properties of steel bars.

Diameter Φ (mm)	Yielding strength f_y_ (MPa)	Ultimate strength f_u_ (MPa)	Modulus of elasticity E (GPa)
8	320	453	200
12	465	707	210
18	465	707	210

### Details of test specimen

**Fig. 1:**
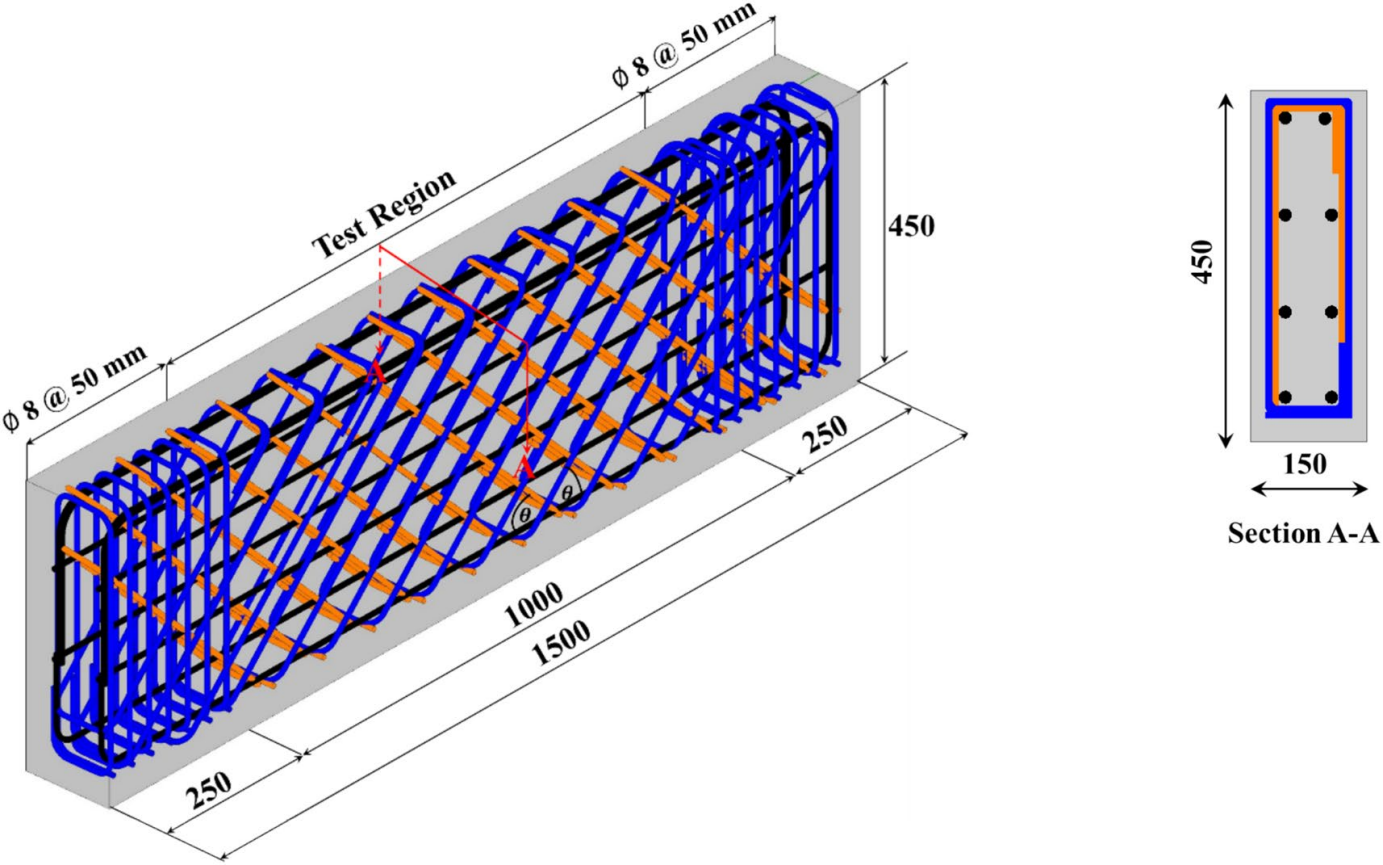
Geometry of the tested deep beams (dimension in mm).

The experimental specimens are intended to investigate the impact of the cross-inclined configuration of HSC deep beams on the pure torsional behavior. Nine rectangular HSC deep beams were designed with a length of 1500 mm and a cross-section dimension of 150 × 450 mm^2^. All of the specimens have the same concrete cover at 20 mm. Each beam was divided into two regions: a pure torsion test region (1000 mm) and a support region (500 mm), as shown in Fig. 1. The two 250 mm extensions at each end were added to simplify the loading system and create torsional moments via eccentric loading arms without reducing specimen span. These dimensions were selected to make the depth-to-ratio equal to 2.22 within the ACI 318–2025’s deep beam classification. In the support region, steel bars were reinforced with 8 mm diameter stirrups spaced at 50 mm to ensure damage occurs in the pure torsion test region. Cross-inclined stirrups are perpendicular as shown in Fig. 1. The variables in these specimens were stirrup spacing (200 – 150 – 100 mm), stirrup type (ordinary closed stirrups – cross-inclined stirrups), inclined stirrup angle (45° – 60°), main longitudinal bars (4 $\varnothing$ 12 – 4 $\varnothing$ 18), and side longitudinal bars (without – 4 $\varnothing$ 12). All these variables, in addition to the ratios of provided longitudinal (${\rho}_{L}$) and transverse (${\rho}_{T}$) reinforcement based on Eqs. (1, 2, and 3) illustrated in Table 2. In Eq. 2, $f$ is equal to 2 for conventional stirrups and 4 for cross-inclined stirrups. The specimens were named such as "DS_i_L_i-ii_" or "DCI_i-ii_L_i-ii_," where the first letter "D" means deep beam. If the second letter is "S," it indicates ordinary closed stirrups, with the following number representing stirrup spacing; if it is "CI," it denotes cross-inclined stirrups, and the subsequent two numbers indicate the stirrups’ inclination angle and spacing, respectively. The third letter "L" denotes longitudinal steel bars, and the next two digits indicate the main and side longitudinal bar diameters, respectively.

**Table 2:** HSC deep beams main parameter details.

Specimen	Longitudinal torsional reinforcement			Transverse torsional reinforcement		
	Main long. bars	Side long. bars	${\rho}_{L}$	Inclination angle	Spacing (mm)	${\rho}_{T}$
DS_200_L_12,12_	4 $\varnothing$ 12	4 $\varnothing$ 12	1.34	90˚	200	0.39
DS_150_L_12,12_	4 $\varnothing$ 12	4 $\varnothing$ 12	1.34	90˚	150	0.52
DS_100_L_12,12_	4 $\varnothing$ 12	4 $\varnothing$ 12	1.34	90˚	100	0.77
DCI_45,200_L_12,12_	4 $\varnothing$ 12	4 $\varnothing$ 12	1.34	45˚	200	1.03
DCI_45,150_L_12,12_	4 $\varnothing$ 12	4 $\varnothing$ 12	1.34	45˚	150	1.37
DCI_45,100_L_12,12_	4 $\varnothing$ 12	4 $\varnothing$ 12	1.34	45˚	100	2.06
DCI_60,100_L_12,12_	4 $\varnothing$ 12	4 $\varnothing$ 12	1.34	60˚	100	1.74
DCI_45,100_L_18,12_	4 $\varnothing$ 18	4 $\varnothing$ 12	2.18	45˚	100	2.06
DCI_45,100_L_12,0_	4 $\varnothing$ 12	–-	0.67	45˚	100	2.06

1 $${\uprho}_{\mathrm{T}}=\frac{{\mathrm{A}}_{\mathrm{T}}{*\uprho }_{0}}{{\mathrm{A}}_{\mathrm{C}\mathrm{P}}*\mathrm{S}}$$

2 $${\uprho}_{0}=f\left(\frac{{h}_{0}}{\mathrm{sin}\theta }+\frac{{b}_{0}}{\mathrm{sin}{\theta}_{top}}\right)$$

3 $${\uprho}_{\mathrm{L}}=\frac{{A}_{L}}{ b*h}$$

### Test set-up and procedure

**Fig. 2:**
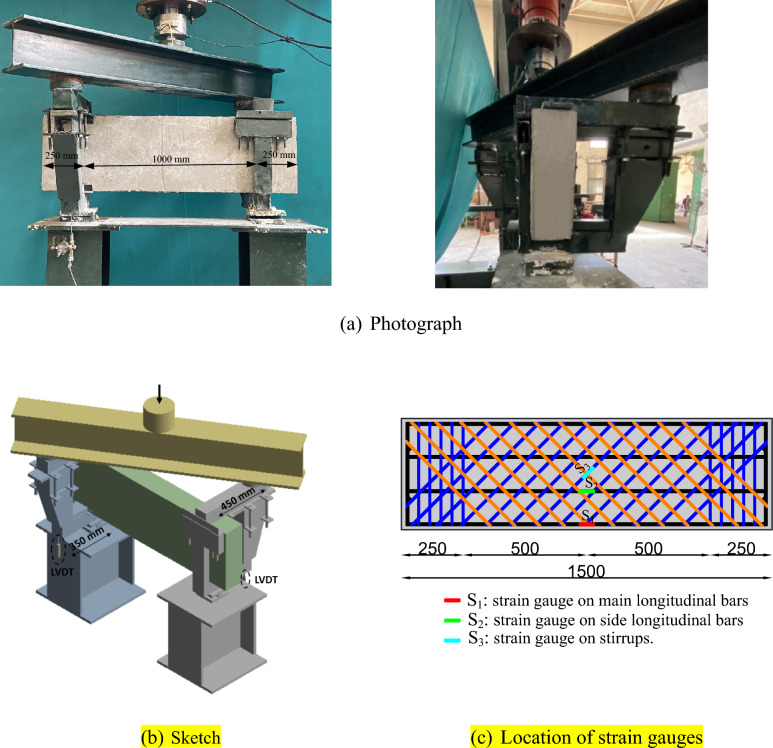
Torsion test set-up.

The test configuration comprises a gravity loading system in which static forces were incrementally delivered using a force-controlled actuator. As illustrated in Fig. 2, a spreader beam was positioned diagonally on two cantilevered arms that were clamped on the specimen to transfer the load to the specimen. The cantilever arms provided the required 450 mm eccentricity. The specimen will therefore experience pure torsion when the jack pushes the spreader beam, as half of the force is transferred to the cantilevered arms. The beam was allowed to move and elongate freely in the longitudinal direction by using two roller supports.

To get pertinent information on the failure mechanisms of the tested beams, the strains of the longitudinal and transverse steel reinforcements were assessed. The locations of the steel strain gauges are shown in Fig. 2(c). The angle of twist was estimated using two linear variable differential transducers (LVDTs). The LVDTs were used under the cantilever arm (at 350 mm from the specimen’s center line) to record the deflection due to the applied load. Load measurements, deformations, and steel strains were continually monitored and documented using a data acquisition system. The test was performed under displacement control circumstances, with the failure of the specimens designated as the stopping criterion. It is important to note that measurements are documented relative to an initial zero state before loading to clearly isolate the influence of the test setup’s self-weight.

## Experimental results and discussions

### Cracking and torsional capacity

**Fig. 3:**
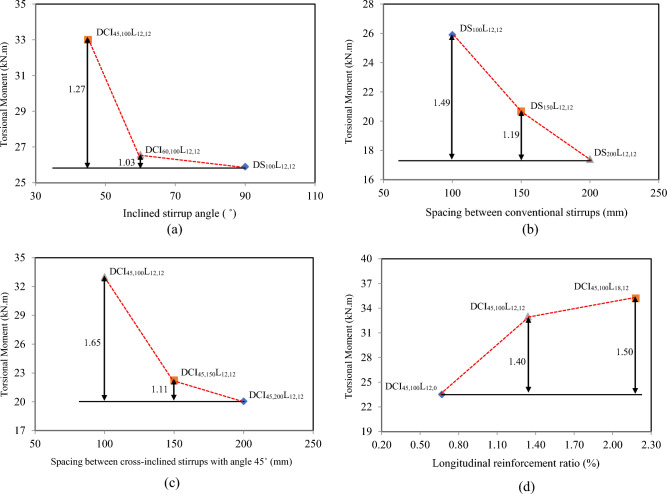
Effect of the tested parameters on the torsional moment capacity.

The cracking and ultimate torsional moments and the associated recorded angles of twist are given in Table 3. The cracking torque varied from 64 to 86% of the ultimate torque. The ultimate torsional capacity is contingent upon variations in the transverse and longitudinal reinforcement ratios. Using inclined stirrups significantly enhances ultimate torque; specifically, when inclined stirrups are used at 60 and 45 degrees, the ultimate torque increases by 3% and 27%, respectively, compared to conventional stirrups, as shown in Fig. 3(a). Which indicates that the ideal inclination angle was 45 degrees, as it was almost perpendicular to the cracks. Reducing the distance between stirrups improves the test beams’ confinement and increases the ultimate torque. For example, compared to the DS_200_L_12,12_ beam, the ultimate torque for DS_150_L_12,12_ and DS_100_L_12,12_ increased by 19 and 49%, respectively. Furthermore, relative to DCI_45,200_L_12,12_, it increased by 11 and 65% for DCI_45,150_L_12,12_and DCI_45,100_L_12,12_, respectively, as illustrated in Fig. 3(b-c). On the other hand, the longitudinal reinforcement has a significant impact on the ultimate torque. Augmenting the longitudinal reinforcement ratio from 0.67 to 1.34 and 2.18%, the torsional capacity increased by 40 and 50%, respectively, as shown in Fig. 3(d).

**Table 3:** Experimental test result.

Specimen	At cracking		At yielding		At ultimate		$\alpha$ (˚)
	T_cr_ (KN.m)	${\theta}_{cr}$(rad/m)	T_y_ (KN.m)	${\theta}_{y}$(rad/m)	T_u_ (KN.m)	${\theta}_{u}$(rad/m)	
DS_200_L_12,12_	14.73	0.0070	17.10	0.033	17.41	0.036	44
DS_150_L_12,12_	17.08	0.0071	20.30	0.033	20.67	0.037	46
DS_100_L_12,12_	19.00	0.0084	25.00	0.047	25.90	0.061	52
DCI_45,200_L_12,12_	17.34	0.0065	19.75	0.017	20.05	0.023	41
DCI_45,150_L_12,12_	17.90	0.0055	20.92	0.012	22.23	0.026	44
DCI_45,100_L_12,12_	22.10	0.0050	27.16	0.011	33.00	0.040	44
DCI_60,100_L_12,12_	20.75	0.0060	25.48	0.025	26.57	0.034	48
DCI_45,100_L_12,0_	19.97	0.0051	23.50	0.019	23.52	0.019	42
DCI_45,100_L_18,12_	22.5	0.0048	28.16	0.001	35.19	0.042	47

### Crack pattern and mode of failure

**Fig. 4:**
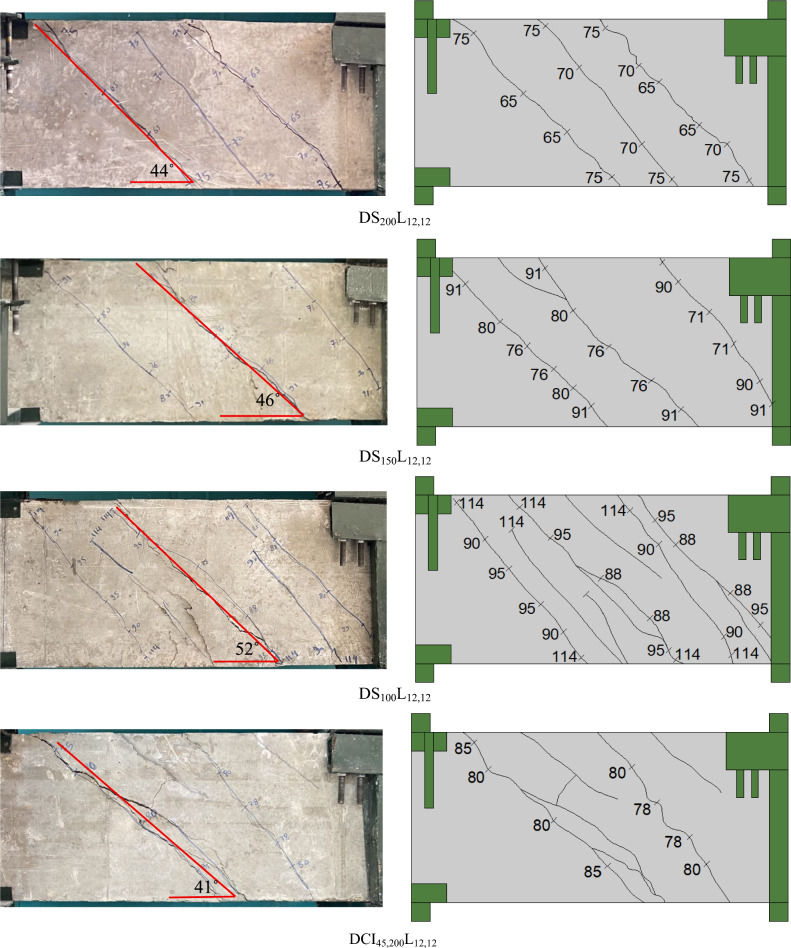

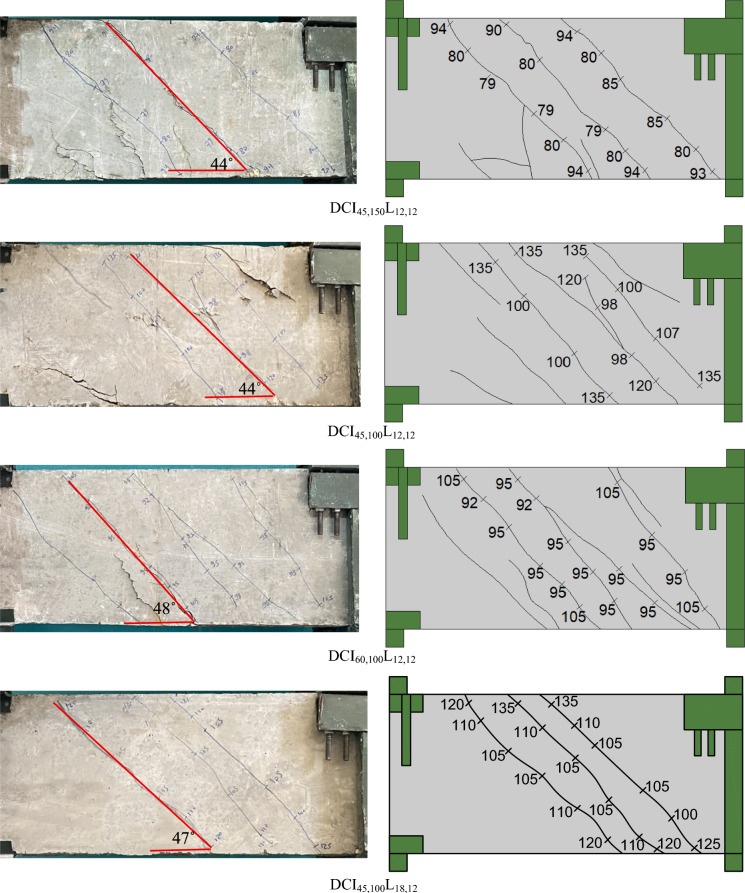

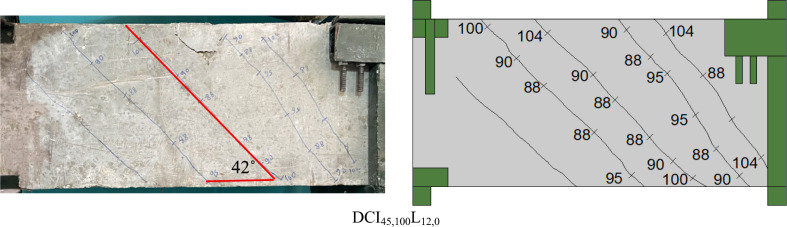
Crack pattern of all tested deep beams.

**Fig. 5:**
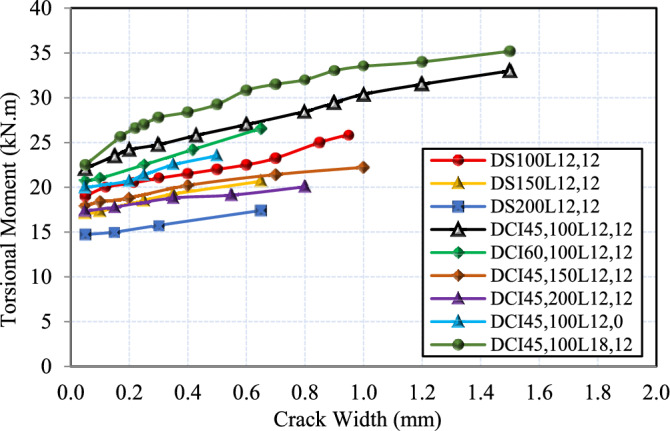
Torsional moment-maximum crack width relationship.

Figure 4 displays photographs of the crack patterns at failure for the tested deep beams. The numbers assigned along the cracks in the photographs represent the torsional load (in kN) (gained from the load cell) near the crack’s termination. The testing proceeded until the specimen failed. Stopping criteria were a major decline in the ultimate capacity (20% of the ultimate torque) associated with large torsional cracks that caused stiffness and structural integrity degradation. Figure 5 shows plots of the measured maximum crack width versus torsional moments. The crack width was measured at each loading step using a special microscope and taken at the first visible crack in the midspan of the specimen. The crack pattern of all investigated beams resembles the same pattern, with only minor differences in the number of cracks and crack width due to the various studied parameters. At angles between 41 and 52 degrees with regard to the beam’s horizontal plane, the first crack appeared at the cracking torque moment. The cracks parallel to the initial crack continued to form in a spiral pattern as the load increased. As the applied torsional moment increased, the crack width increased rapidly. Finally, an inclined crack in the pure torsion region grew wider, causing the tested deep beams to fail. The transverse reinforcement has significant effects on the torsional behavior. It is confirmed from the test findings of Fig. 5 that employing cross-inclined stirrups exhibited a significant cracking behavior in contrast to using conventional stirrups. When compared to the DS_100_L_12,12_ deep beam, the DCI_45,100_L_12,12_ and DCI_60,100_L_12,12_ showed the finest and most evenly distributed cracks. Increasing the distance between the stirrups resulted in fewer spiral cracks, as illustrated in Fig. 4. In addition to increasing crack width. In Fig. 5, the crack width of DS_200_L_12,12_ was somewhat more than that of DS_150_L_12,12_, then DS_100_L_12,12_, and the same was true for DCI_45,200_L_12,12_, DCI_45,150_L_12,12_, and DCI_45,100_L_12,12_, respectively. Furthermore, as Fig. 5 illustrates, increasing the longitudinal ratio enhanced the specimens’ behavior and reduced the crack width.

### Torsional moment versus twist angle curves

**Fig. 6:**
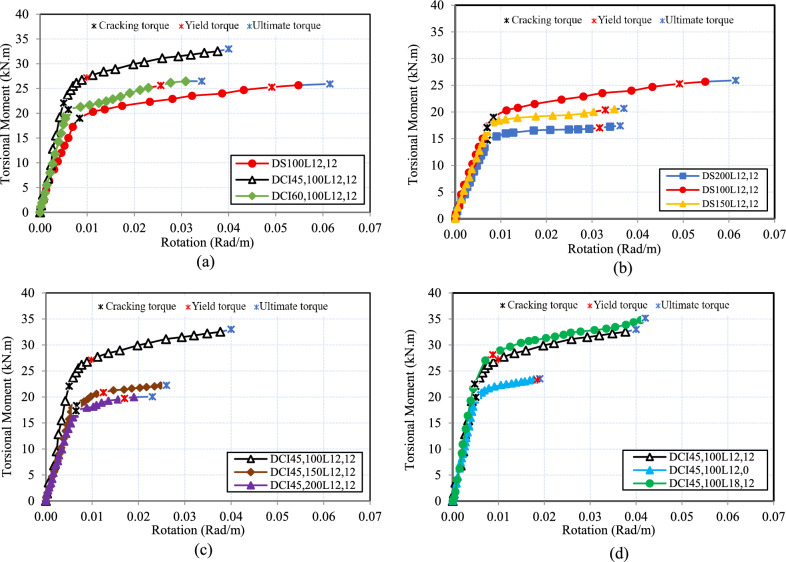
Torsional moment–angle of twist relationship.

Figure 6 illustrates the torque-twist curve for the tested HSC deep beams. First, the curve displays a linear elastic response exhibiting high torsional stiffness prior to cracking, with the concrete section remaining uncracked. Thereafter, the twist of HSC deep beams began to increase swiftly with a slight increase in the torsional moment, marking the beginning of the crack. This phase is distinguished by a decline in stiffness due to the formation of spiral cracks. Finally, the curve attains its maximum capacity. It is confirmed by the test findings in Fig. 6(a) that deep beams with cross-inclined stirrups perform significantly better torsionally than similar beams with conventional stirrups at the same torque. Additionally, it is evident that at the same level of loading, the twist tended to increase as the conventional or spiral reinforcement spacing increased, as shown in Fig. 6(b-c), respectively. Augmenting the ratio of longitudinal bars decreases the angle of twist for the same transverse reinforcement ratio, as illustrated in Fig. 6(d).

### Torsional moment versus strain curves

**Fig. 7:**
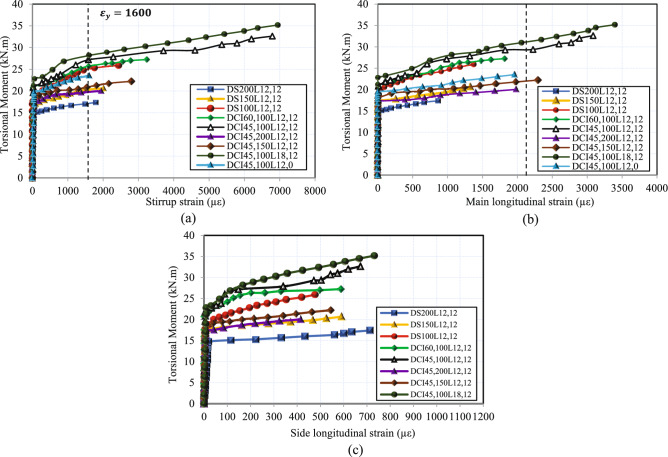
Torsional moment-strain relationship.

The torque versus strain curves for each HSC deep beam’s main longitudinal bar, side longitudinal bar, and stirrup at the midspan are displayed in Fig. 7. The variation of rebar strain and torque for all beams was basically identical. First, the main longitudinal bar, side longitudinal bar, and stirrup all showed minimal and linear strain development. This implies that the torque was mostly resisted by concrete, and the reinforcing bars do not influence the beam’s torsional capacity prior to the initial cracking of the concrete. Once cracking occurs, the strain on reinforcement bars starts to increase immediately. These strains continue to increase, coinciding with the widening of the cracks. As noticed in Fig. 7, the stirrups were the first to yield. This indicates that they were primarily responsible for controlling the first stage of the post-cracking response. Once yielding of the stirrups started, strain increased in the longitudinal reinforcement, specifically in the main longitudinal bars, some of which reached yield, whereas the side longitudinal bars showed lower strains within the elastic range, as seen in Fig. 7(b-c). Cross-inclined stirrups effectively resisted the tensile forces in the beam after cracking, as demonstrated in Fig. 7(a). Their ability to capture cracks was enhanced by their intersection with the diagonal crack, which experienced greater tensile forces during cracking. This effect becomes evident when comparing DS_100_L_12,12_ with DCI_45,100_L_12,12_ and DCI_60,100_L_12,12_; it is clear that 45 degrees is the ideal cross-inclined angle since this angle is perpendicular to the crack. Furthermore, the test findings showed that the effective stirrup strains decreased with decreasing stirrup spacing at the same torque, as shown in Fig. 7(a). The distribution of shear force on additional stirrups intersected by diagonal shear fractures accounts for the reduction in average stirrup strain. Additionally, increasing the area of longitudinal bars reduces the development of strain, as shown in Fig. 7(a).

### Torsional deformation characteristics

Simultaneous investigations of tests for ductility and stiffness were made to ensure a thorough analysis of the entire behavior under torsion for HSC deep beams. Ductility is the ability to withstand deformation after yielding and failure safety. Torsional stiffness is essential during the serviceability and deformation of the structures. Assessing the different factors assures a comprehensive investigation of strength and deformation properties under pure torsion.

#### Torsional ductility index

Torsional ductility refers to a reinforced concrete member’s ability to tolerate considerable inelastic deformation and rotation. It can be determined by the torsional ductility index proposed by Bernardo and Lopes^40,41^. The torsional ductility index is calculated as the ratio of the ultimate angle of twist to the angle of twist at yielding, using the torsional moment-twist response.

4 $${\mu}_{d}=\frac{{\uptheta}_{\mathrm{u}}}{{\uptheta}_{\mathrm{y}}}$$

**Fig. 8:**
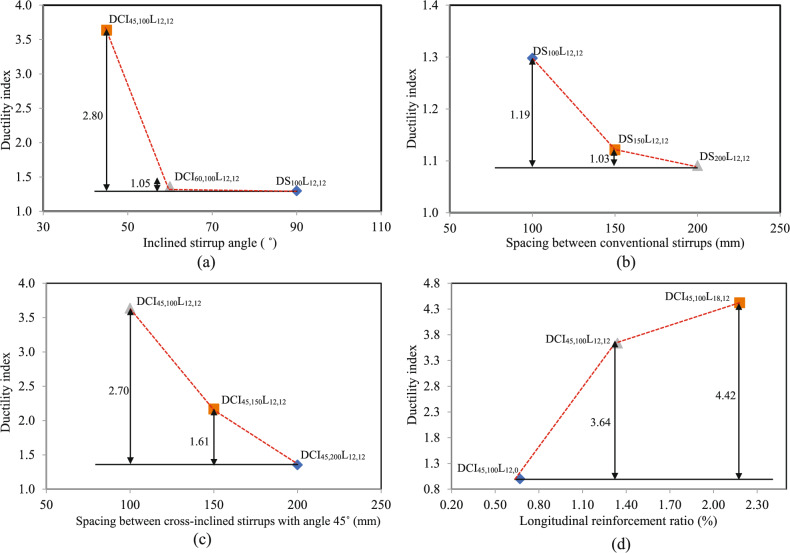
Effect of the tested parameters on the ductility index (${\mu}_{d}$).

Table 4 illustrates the ductility index values for the tested HSC deep beams. The findings indicated that cross-inclined stirrups with 45˚ boosted ductility for deep beams by 2.80 compared to conventional stirrups, as shown in Fig. 8(a). Based on the findings, the torsional ductility was increased by 1.03 and 1.19 for conventional stirrups and by 1.61 and 2.70 for cross-inclined stirrups when the spacing was decreased from 200 to 150 mm and 100 mm, respectively, as shown in Fig. 8(b-c). Because the internal force redistribution is more effective when the stirrup ratio is higher. As a result, the test beam’s surface had reduced average crack width and spacing, which increased overall deformation prior to crack-instability-caused damage. Figure 8(d) depicts the impact of the longitudinal reinforcement ratio on the HSC deep beam’s ductility index. With a similar stirrup ratio, the longitudinal reinforcement ratio increased from 0.67 to 1.34 and 2.18%, and the ductility index increased by 3.64 and 4.42, respectively. It is important to note that the improvement in ductility from increased reinforcement occurs because all specimens are under the optimum reinforcement limit; exceeding this limit is expected to lead to ductility degradation, which is influenced by prior concrete crushing.

**Table 4:** Torsional deformation characteristics test result.

Specimen	Ductility	$\mathrm{s}\mathrm{t}\mathrm{i}\mathrm{f}\mathrm{f}\mathrm{n}\mathrm{e}\mathrm{s}\mathrm{s}$		
	${\upmu}_{\uptheta }$	${\mathrm{K}}_{1}$ (kN.m^2^/rad)	${\mathrm{K}}_{2}$ (kN.m^2^/rad)	$\mathrm{K}$ (%)
DS_200_L_12,12_	1.09	2101.28	92.4	4.40
DS_150_L_12,12_	1.12	2405.63	120.07	4.99
DS_100_L_12,12_	1.30	2661.90	131.18	5.80
DCI_45,200_L_12,12_	1.35	2667.69	164.24	6.16
DCI_45,150_L_12,12_	2.17	3254.55	211.22	6.49
DCI_45,100_L_12,12_	3.64	4402.39	311.607	7.08
DCI_60,100_L_12,12_	1.36	3458.33	207.86	6.01
DCI_45,100_L_12,0_	1.00	3954.46	254.48	6.44
DCI_45,100_L_18,12_	4.42	4687.50	341.13	7.28

#### Torsional stiffness

Torsional stiffness refers to a member’s capacity to withstand twisting under applied torque. Table 4 shows the torsional stiffness of HSC deep beams during loading. The pre-cracking torsional stiffness (K1) represents the stiffness of the test beam during the elastic stage; the torque-twist curve was linear prior to cracking and had the highest value of stiffness, as shown in Table 4. The pre-ultimate torsional stiffness (K2) represents stiffness in the plastic stage, the torque-twist curve was nonlinear, and the tangent slope of the torque-twist curve decreased constantly, as indicated in Table 4. Furthermore, stiffness degradation (K) was used as a comparative indicator for various beam specimens to evaluate the effect of reinforcing arrangement on post-cracking torsional behavior^42^. This attribute made it simple to check the performance of different types of reinforcement in restraining torsion and retaining stiffness after crack formation. The torsional stiffness was calculated from Eqs. (5), (6), and (7).

5 $${K}_{1}=\frac{{\mathrm{T}}_{\mathrm{c}\mathrm{r}}}{{\uptheta}_{\mathrm{c}\mathrm{r}}}$$

6 $${K}_{2}=\frac{{\mathrm{T}}_{\mathrm{u}}- {T}_{cr}}{{\uptheta}_{\mathrm{u}}- {\theta}_{cr}}$$

7 $$K=\frac{{\mathrm{K}}_{2}}{{\mathrm{K}}_{1}}$$

**Fig. 9:**
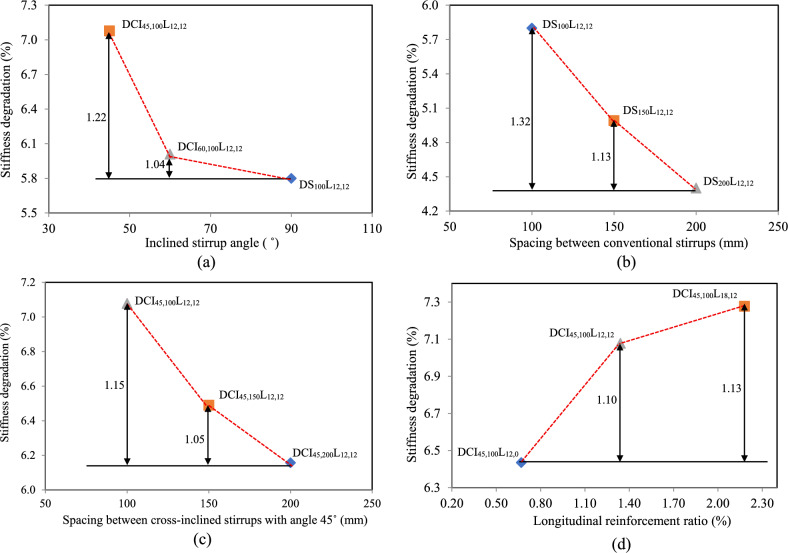
Effect of the tested parameters on the stiffness degradation (K).

The results indicated that employing cross-inclined stirrups with 45˚ increased torsional stiffness by 22%. This improvement was clearly demonstrated in the stiffness degradation values provided in Fig. 9(a). Furthermore, when the spacing of conventional and cross-inclined stirrups was decreased from 200 to 150 mm and 100 mm, the K of HSC deep beams significantly increased, as shown in Fig. 9(b-c). The stiffness deterioration of HSC deep beams improved marginally when the longitudinal reinforcement ratio increased, as seen in Fig. 9(d).

## Numerical investigation

A three-dimensional nonlinear finite element model is proposed to examine the capability to capture the observed torsional behavior in the experiment of HSC deep beams reinforced by cross-inclined stirrups. For this model, the computer program ABAQUS^43^ is used.

### Numerical data input

#### Element description

Two primary types of elements were used to produce a 3D FE model of HSC deep beams reinforced with cross-inclined stirrups: solid elements and truss elements.

- Solid element

**Fig. 10:**
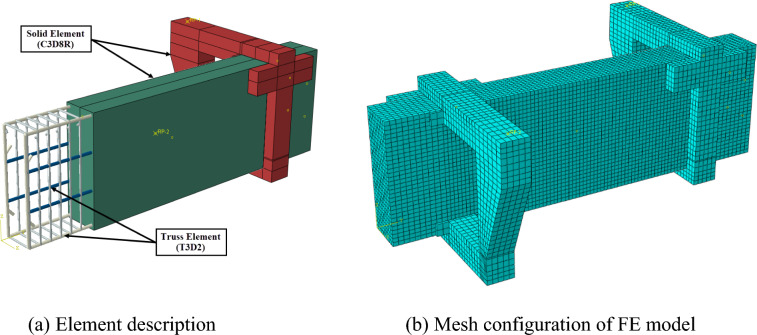
Element and mesh configuration of FE model.

Concrete deep beams and cantilever arms are modeled using Solid C3D8R, which has eight nodes with three transitional degrees of freedom. The selection of C3D8R is based on its capacity to specify boundary conditions and specify the concrete property, as well as the contact face. Figure 10(a) shows the parts created. The mesh element was 15 mm × 15 mm × 15 mm for the tested region and 20 mm × 20 mm × 20 mm for the +support region and cantilever arms, as shown in Fig. 10(b). This mesh size was chosen in accordance with a sensitivity analysis to adjust the model according to alternative mesh sizes from 10 to 30 mm for the tested region and the support region, with results compared against experimental data. Thus, the adopted mesh sizes ensure a balance between accuracy and computational efficiency.

b. Truss element

**Fig. 11:**
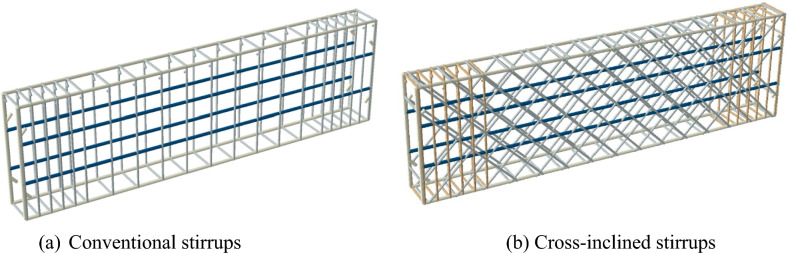
Different reinforcing configurations of the FE model.

The T3D2 element was determined for modeling reinforcement bars and stirrups, which can only carry compressive or tensile forces. Figure 11 depicts the different reinforcing cages, which were simulated as embedded elements in a concrete block. The steel reinforcement truss element was 15 mm in mesh size.

#### Material modelling

**Fig. 12:**
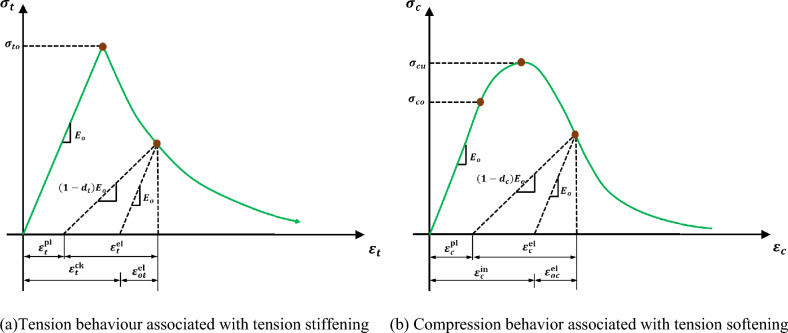
Concrete damaged plasticity relationships.

In this paper, a stress-based analysis was conducted for identifying the initiation and development of cracks in casting high-strength concrete. The stress-based formulation is consistent with the Concrete Damaged Plasticity (CDP) model in Abaqus^43^, which identifies tensile cracking when the maximum primary stress criterion or tensile cracking criteria are violated. With the smeared cracking approach, this formulation allows one to accurately locate regions and orientations that are susceptible to cracking with high geometric accuracy. Nonlinear material behavior was integrated using the Concrete Damaged Plasticity (CDP) model for concrete and elastic–plastic hardening for reinforcement in both tension and compression. For concrete subjected to compression, its stress–strain curve starts off linearly, reaches a strain-hardening region until the peak point, and ends with a softening region. The tensile properties consider fracture energy to reflect the stiffening loss of post-cracked specimens, as shown in Fig. 12. The above procedure was commonly adopted in modeling reinforced concrete numerically in past studies^44^. The elastic properties of concrete are estimated to have a density of 2200 kg/m^3^, an elasticity modulus of 41.97 GPa, and a Poisson’s ratio of 0.22. The parameters of the damaged plasticity model of concrete are displayed in Table 5. A perfect bond between concrete and reinforcement was assumed. Reinforcing steel is supposed to have a density of 7859 kg/m^3^, an elasticity modulus of 210 and 200 GPa for high tensile and mild steel, respectively, and a Poisson’s ratio of 0.30.

**Table 5:** Concrete damaged plasticity (CDP) parameter.

Parameter	dilation angle	eccentricity	f_bo_/f_co_	K	viscosity
Value	43	0.12	1.36	0.67	0.00001

#### Boundary conditions

**Fig. 13:**
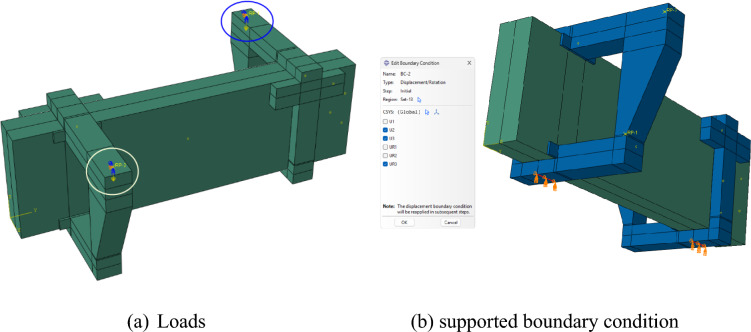
Simply supported boundary condition of model.

The load was applied on the reference points, which were restricted from translating and rotating in all directions except translation in U2, as illustrated in Fig. 13(a). In addition, the experimental support conditions were replicated using appropriate boundary conditions. Roller supports were defined under each cantilever arm, as shown in Fig. 13(b).

### Comparison between FEM and experimental test results

**Fig. 14:**
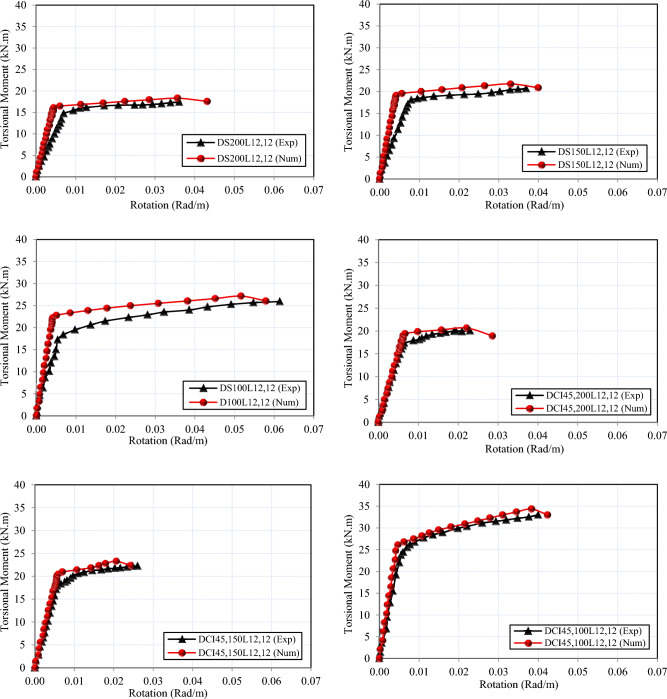

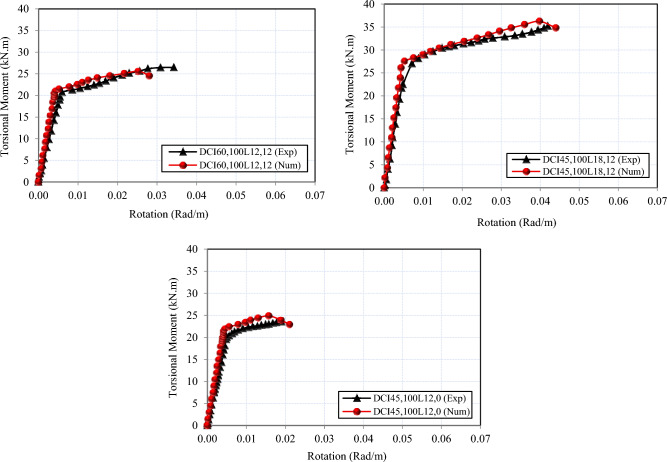
Numerical and experimental torsional moment–angle of twist curves for all tested deep beams.

**Fig. 15:**
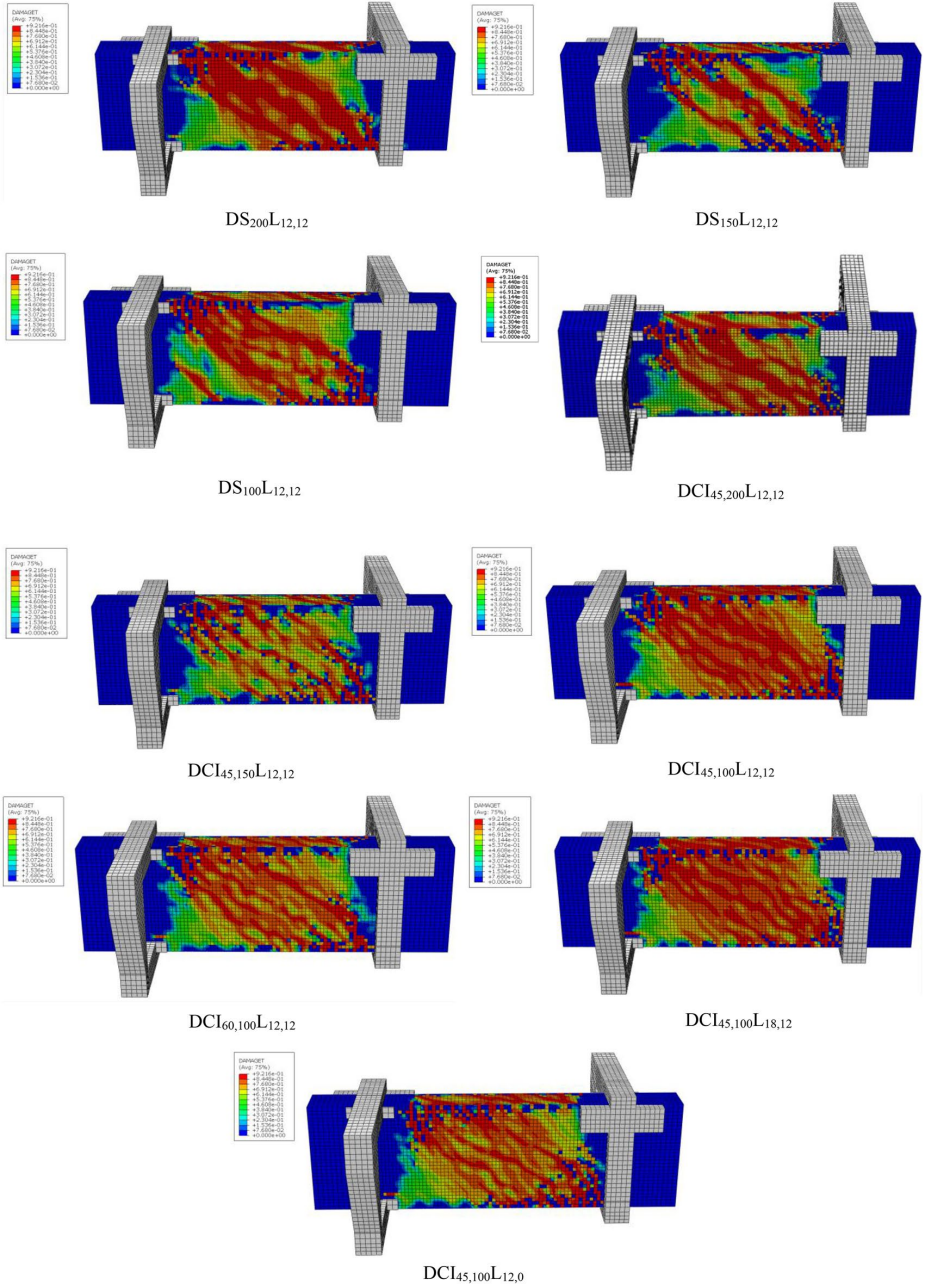
FE crack pattern of all tested deep beams.

The values of all the tested specimens using finite element modeling exhibit strong concordance with the experimental findings. As demonstrated in Table 6, the average difference between finite element (FE) and experimental data was around 90% for the cracking torsional moment and 97% for the ultimate torsional moment. Furthermore, Fig. 14 provides the numerical and experimental torsional moment–angle of twist relationships of the studied deep beams. These evaluations demonstrated that the twist relationships of HSC deep beams reinforced with cross-inclined stirrups are well predicted by the proposed model. Figure 15 indicates that the failure patterns of all FE models for all investigated specimens were identical to that of the same beams investigated experimentally in Fig. 4. As a result, the comparison was considered very effective. The aforementioned findings confirm that the suggested FE models precisely depict the real behavior of HSC deep beams under pure torsion, as the numerical results’ values are strikingly similar to the experimental results.

**Table 6:** Comparisons between experimental and numerical results.

Specimen	T_cr,Num._ (KN.m)	T_cr,exp_ / T_cr,Num_	T_u,Num._ (KN.m)	T_u,exp_ /T_u,Num_
DS_200_L_12,12_	16.11	0.91	18.31	0.95
DS_150_L_12,12_	19.14	0.89	21.75	0.95
DS_100_L_12,12_	22.80	0.83	27.15	0.95
DCI_45,200_L_12,12_	19.42	0.89	20.66	0.97
DCI_45,150_L_12,12_	20.02	0.89	23.27	0.96
DCI_45,100_L_12,12_	26.10	0.85	34.34	0.96
DCI_60,100_L_12,12_	20.98	0.99	25.59	1.04
DCI_45,100_L_12,0_	21.41	0.93	24.90	0.94
DCI_45,100_L_18,12_	26.10	0.86	36.24	0.97

## Ultimate torque proposed model

This study demonstrated that using cross-inclined stirrups in deep beams significantly improved torsional capacity. As a result, the stirrup inclination angle should be accounted in the design equations. To anticipate the torsional capacity, the torsional behavior of a standard RC beam is divided into two regions: the elastic until the first cracking part and the after-cracking part. The differences in response in these places reflect the characteristics of the load-resisting system in each instance.

### Cracking torque

The provisions for calculating the cracking torsional moment could be calculated using ACI 318–2025^6^. Equation (8) illustrates the cracking torque load-calculating formula, which is based on thin-walled tube theory. The cracking torsional moment depends on the concrete strength and cross-section. Meanwhile, the influence of steel reinforcement is ignored due to their low stress before cracking.

8 $${\mathrm{T}}_{\mathrm{c}\mathrm{r}}=0.33\frac{{A}_{cp}^{2}}{{ P}_{c} }\sqrt{{f}_{c}}$$

### After cracking torque

Many academics have acknowledged the softened space truss model^18^ as an approach for calculating ultimate torque in RC beams with longitudinal bars and stirrups. The space truss analysis is predicated on the concept that an internal torque generated by the shear flow q, which develops in the center of a shear flow zone with an effective wall thickness t_d_, resists the exterior torsional moment T. Furthermore, using the stress equilibrium, the following known relationships are produced for calculating the longitudinal and the transverse torsional strengths, T_L_ and T_T_^18,45^ in the case of conventional stirrups. The inclination of the diagonal compression struts (cracking angle $\alpha$) is equal to 45° according to ACI 318–2025^6^. Besides that, this angle matches the average crack angles found in the experiment.

9 $${\mathrm{T}}_{\mathrm{L}}=\frac{ 2{A}_{L}{f}_{L}{A}_{o}}{{P}_{o}}\text{ tan}\alpha$$

10 $${\mathrm{T}}_{\mathrm{T}}=\frac{ 2 { A}_{T} {f}_{T} {A}_{o} }{S}\mathrm{cot}\alpha$$

11 $${A}_{o}=0.85\left(\frac{{h}_{o}}{\mathrm{sin}\theta }*\frac{{b}_{o}}{\mathrm{sin}{\theta}_{top}}\right)$$

**Fig. 16:**
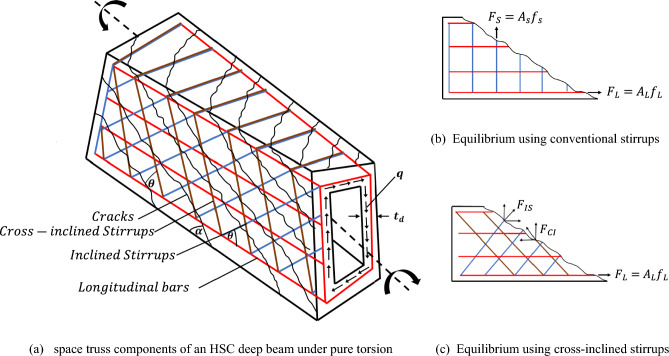
Torsional analysis based on space truss theory.

Chalioris and Karayannis^34^ proposed a model for calculating ultimate torque using the inclination angle of spiral stirrups depending on the space truss model. In this paper, the ultimate torque of HSC deep beams reinforced by longitudinal bars and cross-perpendicular inclined stirrups was calculated using a formula based on Chalioris and Karayannis. In the instance of specimens with cross-inclined stirrup reinforcement as transverse steel reinforcement, the following equations can be acquired from the equilibrium of a section of the vertical wall of the beam, as displayed in Fig. 16(c). The force equilibrium along the longitudinal direction:

12 $$q{p}_{o}=\left({{A}_{L}f}_{L}+{A}_{IS}{f}_{Is}cos\theta -{A}_{CI}{f}_{CI}cos\theta \right) tan\alpha$$

The force equilibrium along the transverse direction:

13 $$qs=\left({A}_{IS}{f}_{IS}sin\theta +{A}_{CS}{f}_{CS}sin\theta \right) cot\alpha$$

Substituting the shear flow (q) in Eqs. (12) and (13) with the equation of Bredt:

14 $$q=\frac{T}{ 2{A}_{o}}$$

It can be derived:

15 $${\mathrm{T}}_{\mathrm{L}}=\frac{ 2{A}_{o} \left({{A}_{L}f}_{L}+{A}_{IS}{f}_{Is}cos\theta -{A}_{CI}{f}_{CI}cos\theta \right) }{{P}_{o}}\text{ tan}\alpha$$

16 $${\mathrm{T}}_{\mathrm{T}}=\frac{ 2 {A}_{o}\left({A}_{IS}{f}_{IS}sin\theta +{A}_{CI}{f}_{CI}sin\theta \right) }{S}\mathrm{cot}\alpha$$

Finally, the following formula can be used to ascertain the ultimate torque capacity for deep beams reinforced by cross-inclined stirrups:

17 $${T}_{u}={T}_{cr}+min\left\{\begin{array}{c}{T}_{T}\\ {T}_{L}\end{array}\right.$$

### Comparison with experimental results

The experimental data and the formula’s calculated results were compared in order to validate the calculation’s accuracy for the proposed formula. As demonstrated in Table 7, the average difference between the proposed and experimental data was around 92% for the ultimate torsional moment, which indicated that the formula proposed in this paper can predict the ultimate torque of deep beams using cross-inclined stirrups accurately.

**Table 7:** Comparisons between experimental and theoretical results.

Specimen	T_cr,Theo._ (KN.m)	T_cr,exp_ / T_cr,Theo._	T_u, Theo._ (KN.m)	T_u,exp_ /T_u,Theo_
DS_200_L_12,12_	10.69	1.85	16.86	1.03
DS_150_L_12,12_	10.69	1.60	18.92	1.09
DS_100_L_12,12_	10.69	1.38	23.03	1.12
DCI_45,200_L_12,12_	10.69	2.07	23.03	0.87
DCI_45,150_L_12,12_	10.69	1.94	27.14	0.82
DCI_45,100_L_12,12_	10.69	1.62	35.37	0.93
DCI_60,100_L_12,12_	10.69	1.67	35.37	0.75
DCI_45,100_L_12,0_	10.69	1.87	32.61	0.72
DCI_45,100_L_18,12_	10.69	2.10	35.37	0.99

## Conclusion

Experimental and numerical studies are conducted to investigate the behavior of high-strength concrete (HSC) deep beams with cross-inclined stirrups as transverse reinforcement under pure torsion.

- By using cross-inclined stirrups, the torsional behavior of HSC deep beams was improved considerably. The optimal value for the inclination angle was 45 degrees, as it was almost perpendicular to the cracks. The torsional capacity was augmented by 27%. Reducing the spacing of conventional stirrups resulted in a 19% and 49% enhancement in torsional capacity. While decreasing the distance between cross-inclined stirrups, the maximum torsional capacity increases by 11% to 65%. The torsional strength was enhanced by boosting the longitudinal reinforcement ratio up to 50%.
- The arrangement of reinforcement affects the mode of failure and the crack pattern. Cracks were more distributed when cross-inclined stirrups were used. Cracks were more numerous and narrower when stirrups were spaced closer together. Increasing the longitudinal ratio enhanced the specimens’ behavior and reduced the crack width.
- Deep beams with cross-inclined stirrups showed reduced angles of twist at the same levels of torsion, proving improved torsional stiffness. Cross-inclined stirrups effectively resisted tensile stresses, leading to the efficient use of steel reinforcement.
- Cross-inclined stirrups significantly improved torsional ductility up to 2.80 compared to conventional closed stirrups. Furthermore, the torsional ductility was increased by 1.03 and 1.19 for conventional stirrups and by 1.61 and 2.70 for cross-inclined stirrups when the spacing was decreased, because the internal force redistribution is more effective when the stirrup ratio is higher. In addition, increasing the longitudinal reinforcement ratio led to an enhancement of the ductility index by 3.64 and 4.42.
- Cross-inclined stirrups greatly enhanced stiffness by up to 22% when compared to conventional closed stirrups. Additionally, reducing the spacing between conventional and cross-inclined stirrups significantly increased the stiffness of HSC deep beams. The stiffness deterioration of HSC deep beams improved up to 13% when the longitudinal reinforcement ratio increased.
- The non-linear finite element model, which was developed by ABAQUS, has been found to accurately simulate the torsional behavior of the tested deep beams in terms of torsional capacity, torque-twist relationship, and crack pattern; the numerical results obtained by the finite element model were found to match the experimental results. Hence, the reliability of the finite element model could be confirmed for predicting the torsional behavior of HSC deep beams reinforced by cross-inclined stirrups.
- The theoretical model based on ACI 318–2025 accurately predicted the torsional capacity of deep beams under pure torsion, with an average agreement rate of 92%.

Finally, this paper is conducted on HSC deep beams with a cross-section of 150 × 450 mm, a clear span of 1000 mm (L/h = 2.22), and a compressive strength of 91.7 MPa. The findings cannot be extended to deep beams whose geometrical shapes, materials, or span-to-depth ratios are different. The findings are confined to particular angles and spacing of cross-inclined stirrups; hence, trends of cross-inclined stirrups should be considered accordingly. The study involved pure torsion, whereas in actual structures, there are other forces such as bending and shear that could make a considerable difference to the structural behavior. The findings are relevant for similar deep beam configurations; however, more research is necessary for wider relevance.

## Data Availability

All data generated or analyses during this study are included in this published article.

## Abbreviations

${A}_{CI},{A}_{IS}$: Area of one legged cross-inclined and inclined stirrups, mm^2^
${\mathrm{A}}_{\mathrm{C}\mathrm{P}}$: Cross-sectional area of the HSC deep beam, mm^2^
${A}_{L}$: Area of longitudinal reinforcement, mm^2^
${A}_{o}$: Area enclosed by the centerline of shear flow path, mm^2^
${\mathrm{A}}_{\mathrm{T}}$: Area of one-legged stirrup, mm^2^
$b,h$: The width and height of the tested beams, mm
${{b}_{0},h}_{0},$: The width and height of the area enclosed by the centerline of the stirrup, mm
${d}_{c}$, ${d}_{t}$: The compression and tensile damage property
${E}_{o}$: The modulus of elasticity of concrete, GPa
${f}_{c}$: The compressive strength of HSC, MPa
${{f}_{CI},f}_{Is}$: The yield strength of cross-inclined and inclined stirrups, MPa
${f}_{L }, {f}_{T}$: The yield strength of longitudinal and transverse reinforcement, MPa
$\mathrm{K}$: Stiffness degradation (pre-ultimate to pre-crcking torsional stiffness), %
${\mathrm{K}}_{1}, {K}_{2}$: The pre-cracking and pre-ultimate torsional stiffness), kN.m^2^/rad
P_c_: The perimeter of HSC deep beam cross-section, mm
${P}_{o}$: Perimeter of centerline of the shear flow in space truss analysis, mm
S: The stirrup spacing of the tested beams, mm
T_cr_, T_y_, T_u_: Cracking, yielding and ultimate torsional moment, kN.m
T_cr,Num_, T_u,Num_: The numerical cracking and ultimate torsional moment, kN.m
T_cr,Theo._, T_u,Theo._: The theoretical cracking and ultimate torsional moment, kN.m
$\alpha$: Diagonal compression struts angle, ˚
${\theta}_{cr}$, ${\theta}_{y}$, ${\theta}_{u}$: Cracking, yielding and ultimate twist, mm
${\varepsilon}_{c}^{el}$, ${\varepsilon}_{t}^{el}$: The compressive and tensile elastic strain, mm/mm
${\varepsilon}_{c}^{in}$: The compressive inelastic strain, mm/mm
${\varepsilon}_{c}^{pl}$, ${\varepsilon}_{t}^{pl}$: The compressive and tensile plastic strain, mm/mm
${\varepsilon}_{oc}^{el}$: The elastic strain corresponding to ${\sigma}_{co}$, mm/mm
${\varepsilon}_{ot}^{el}$: The elastic strain corresponding to ${\sigma}_{to}$, mm/mm
${\varepsilon}_{t}^{ck}$: The tensile cracking strain, mm/mm
$\theta$, ${\theta}_{top}$: The front and top inclination angles of stirrups, ˚
${\mu}_{d}$: Ductility index (ultimate to cracking twist ratio)
${\rho}_{L}$, ${\rho}_{T}$: Longitudinal and transverse reinforcement ratio, %
${\uprho}_{0}$: The perimeter of closed stirrups or cross-inclined stirrups, mm
${\sigma}_{co}$: The yield compressive stress, N/mm^2^
${\sigma}_{cu}$: The ultimate compressive stress, N/mm^2^
${\sigma}_{to}$: The tensile stress of concrete at failure, N/mm^2^
